# 96-well plate-based aggregometry

**DOI:** 10.1080/09537104.2018.1445838

**Published:** 2018-03-15

**Authors:** Melissa V. Chan, Paul C. Armstrong, Timothy D. Warner

**Affiliations:** Centre for Immunobiology, The Blizard Institute, Barts and The London School of Medicine and Dentistry, Queen Mary University of London, London, UK

**Keywords:** Light transmission aggregometry, platelet aggregometry, 96-well plate aggregometry

## Abstract

While there are many bench and bedside tests to assess platelet reactivity, *ex vivo* light transmission aggregometry (LTA) remains the gold standard. LTA, however, is expensive, time-consuming and requires dedicated equipment and staff, making it impractical in many situations. In addition, there is significant variability between data generated at different testing sites meaning that tests often need to be repeated if a patient is transferred to the care of a different hospital. As such, there is clearly an unmet need for standardization of platelet testing.

Using the principles of LTA, aggregometry can be conducted in 96-well plates with readings being made in a standard plate reader. This approach allows for the assessment of multiple concentrations of agonists, since the volume of platelets required for each test is significantly lower than for LTA. Furthermore, the lyophilization of a set panel of agonists to a 96-well plate to produce a stable assay substrate allows the production of portable, standardized plates that can be used to generate reproducible tests at multiple sites.

In this review, we will discuss the methods and uses of 96-well plate aggregometry for both research and the clinic.

## The principles of platelet aggregometry

**Figure 1.**
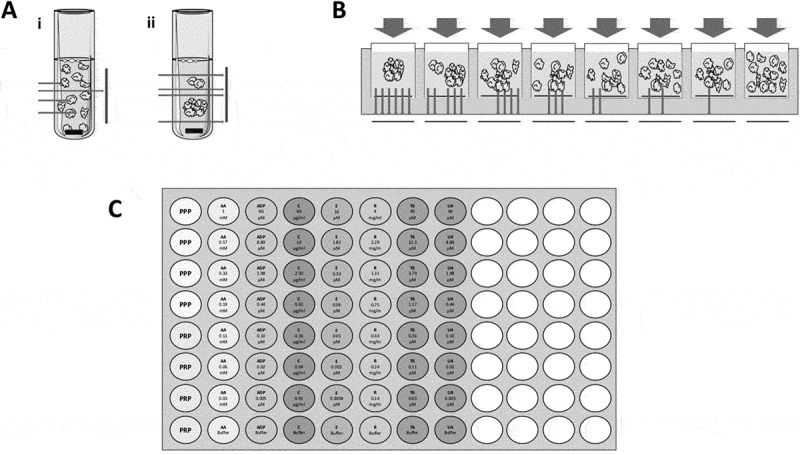
A schematic of (A) traditional light transmission aggregometry (LTA) in the (i) absence and (ii) presence of a platelet agonist as measured by light transmission. When a platelet agonist is added, an aggregate forms and increased transmission of light is detected giving high % aggregation. (B) 96-well plate aggregometry with decreasing concentrations of agonist from left to right. In a plate reader, light absorbance is detected. When an aggregate is formed, less light is absorbed, giving high % aggregation. Conversely, when a low concentration of agonist is added, more light is absorbed, leading to low % aggregation. C) The layout of an Optimul plate with seven concentrations of the platelet agonists AA, ADP, collagen, epinephrine, ristocetin, TRAP-6 amide and U46619 lyophilized onto the plate. % aggregation is determined by control PRP and PPP wells and measured by absorbance in a plate reader. This figure was produced using Servier Medical Art (http://www.servier.com).

The current gold standard for the *in vitro* testing of platelet reactivity is light transmission aggregometry (LTA), developed by Born in the 1960s (1–3). LTA is based upon the principle that light passes more easily through a clear than a turbid solution. Centrifugation of blood can be used to pellet red and white blood cells and so produce a suspension of platelets in plasma, commonly termed platelet-rich plasma (PRP). Further centrifugation of PRP to pellet the platelets results in the generation of platelet-poor plasma (PPP). To characterize platelet aggregation, the change in light transmission of PRP held in a cuvette and mixed by a magnetic stir bar can be observed. In simple terms, as aggregates form, the turbidity of the solution is reduced and this is followed by a photosensor that detects the level of light passing through the solution from a light source. The % aggregation can be subsequently calculated by defining the transmission of light through PRP as being 0% aggregation and the transmission of light through PPP as being 100%, or maximum, aggregation (Figure 1A).

LTA has many advantages. It provides a way to measure platelet function in patients with bleeding disorders (4) and also to assess the efficacy of anti-platelet therapies (5). In addition, the two phases of platelet aggregation (a primary activation and secondary potentiation) as well as disaggregation can be visualized in real time, which are critical in understanding the effects of antagonists or weak agonists.

Despite these advantages, LTA also has a number of logistical drawbacks. Conducting extensive platelet function analysis requires a relatively large volume of blood, since around 200–250 μl of PRP is needed for each condition tested. LTA is also time consuming, since at least 5 minutes is required to run each test, and commercially available aggregometers have a maximum of 8 channels (more often 2 or 4) meaning that only a limited number of tests can be performed in parallel. As well as requiring expensive and dedicated aggregometers, the technique is labour-intensive and requires specialist training of the individual running the tests. Furthermore, though there are clear International Society on Thrombosis and Haemostasis (ISTH) guidelines for the use of various platelet agonists and their concentrations, adherence to these guidelines is poor and varies with local practice and regulations (6,7). Taking together these variables in assay methodology result in data that is very hard to compare and/or share between different sites and operators. The 96-well plate-based platelet aggregometry has been developed to address these issues.

## 96-well plate aggregation

Established in the 1990s, 96-well plate is based on the same principles as LTA but with some modifications. In this assay, PRP is generally added to agonist containing wells of a 96-well plate. The entire plate is shaken by vortex or plate shaker and light absorbance is measured as a surrogate for platelet aggregation, rather than light transmission in LTA. Again, % aggregation is calculated based on the absorbance of PPP and PRP controls (Figure 1B). Incorporating replicates of PPP and PRP to provide reliable controls, this plate-based assay allows for up to 90 different experimental wells to be assayed within one ‘run’ (8,9) making it a far more high-throughput and efficient method than LTA.

Since the first description of 96-well platelet aggregometry, there have been many permutations and, therefore, many variations in the method (8). Here, we discuss these various approaches, compare them to LTA and so outline the bases for standardization.

### Agonists and PRP

**Figure 2.**
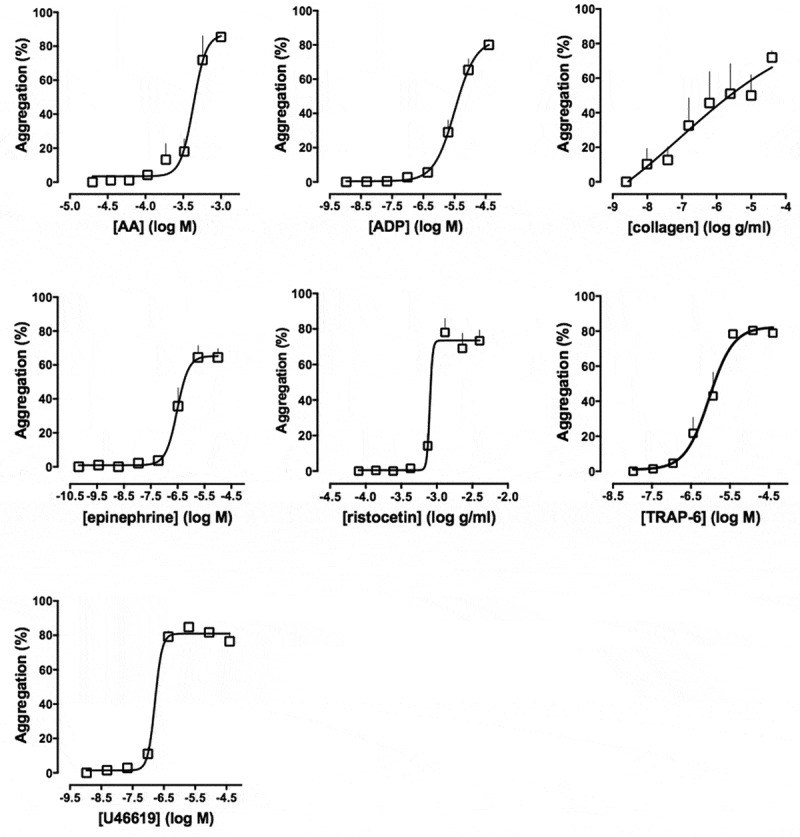
Concentration–response curves of platelet aggregation in response to: AA (0.03–1 mM), ADP (0.005–40 μM), collagen (0.01–40 μg/ml), epinephrine (0.0004–10 μM), ristocetin (0.14–4 mg/ml), TRAP-6 amide (0.03–40 μM) and U46619 (0.005–40 μM) on Optimul plates after 5 minutes of mixing (1200 rpm) at 37°C. Data are shown as mean ± SEM, *n* = 6. Adapted from *Chan et al*. (18).

As with LTA, there are variations between the agonists and the agonist concentrations studied by different research groups. Most groups have studied aggregation in response to ADP, collagen and thrombin receptor-activating peptide (TRAP)-6 (or thrombin in washed platelets), while others have also investigated the effects of epinephrine, serotonin, the thromboxane (Tx) A_2_ mimetic U46619, and ristocetin (10–13). There is little consensus from these various groups with regard to the specific concentrations of agonists to be used. The increased capacity of the 96-well plate, relative to LTA, means that a wide range of the agonist concentrations can be used that encompasses all those reported previously. Example concentration-response curves are shown in Figure 2.

Another source of difference has been the order in which PRP and agonists are added to the wells on the plate. In LTA, agonist is added to PRP which allows for pre-incubation of PRP and pre-agonist readings of light transmission. Transferring this approach to 96-well plate aggregometry introduces inconsistencies between rows on the plate. For example, platelets express P-selectin directly upon contact with agonists, even in static conditions, so speed of pipetting across the plate is critical (14). These concerns may be bypassed by automatically injecting agonists into the wells (15). The alternative is that the different concentrations of agonists can be pipetted into the wells of the plate before the addition of PRP. This method is not as time-sensitive because it allows for the addition of PRP to all wells using a reagent reservoir and electronic or manual multichannel pipette within a few seconds; i.e. in this approach, all wells receive the same solution unlike the complication of adding multiple agonists at multiple concentrations to PRP already present in the plate (11,16,17).

Further considerations include the type of anti-coagulant used to collect blood and also whether PRP is adjusted to normalize platelet count or not, which we have touched upon previously (18). Indeed, we have shown that platelet concentrations of less than 3 × 10^8^/ml cannot be assayed using the 96-well plate method. Since both anti-coagulant and platelet count will affect both LTA and 96-well aggregometry, these are beyond the scope of this review.

### Assay volume

The obvious advantage of the 96-well plate method is the reduction in volume of PRP, and therefore blood, required for any test. Since the wells in most conventional 96-well plates can only hold a maximum of 300 μl, this limits the assay volume. In the original description of the method, Fratantoni *et al*. used 135 μl PRP with 15 μl agonist (8). Krause *et al*., studying the effects of glycoprotein IIb/IIIa antagonists, and Maayani *et al*. also used an assay volume of 150 μl, albeit with different ratios of PRP to agonist (10,16). A few groups have used an assay volume of more than 150 μl (15,17,19), but most have reduced volumes of PRP or washed platelets to around 100 μl (9,11,20–22). As a result, for the same volume of blood collected from a patient, a greater selection of agonists and concentrations may be studied using this assay that using LTA.

### Agitation method

A major methodological difference between reports of 96-well plate-based aggregation is the nature or type of shaking used to mix the PRP and agonists. Some used vortexing (9), some used plate shakers and some used the shakers present within 96-well plate readers. These shakers come in different varieties, e.g. linear (8,16,23), orbital (17,21) and kinetic (10,11,15). In LTA, a stir bar is present in each sample which creates a simple reproducible vortex in the cuvette whereas the internal shakers in plate readers are hugely variable. For instance, the shaker used by the Fratantoni group had an amplitude of 1.3 mm at 1360 rpm, whereas the shaker for Peace *et al*. was a 0.1 mm orbit at 1000 rpm (8,17). Since the physical forces exerted on platelets are a key part of activation pathways, it is important that this variable is standardized between assays (24). While this can be readily achieved with LTA, this cannot be done with plate readers where the mixing function is generally a fixed factory installed option. A further variation between research groups is the duration of shaking, reports include bursts of intermittent shaking for up to 18 minutes or one continuous shake for 5 minutes (17,25). We have previously published traces of absorbance measured every 15 seconds following shaking after agonist stimulation over 16 minutes (12). Since this is slower than LTA, we have subsequently utilized a stand-alone high-speed shaker that provides uniform mixing through the production of vortices in each well of the plate and generates more rapid platelet responses. This approach allows 96-well plate aggregometry to more closely mimic the mixing seen in LTA with a magnetic stirrer bar. Decoupling the mixing of the assay from the reading of the assay allows for any 96-well plate reader to be used to measure platelet aggregation following the purchase of a standardized mixer (25).

The temperature at which the assays are conducted has also varied. The majority of 96-well plate aggregometry has been conducted at 37°C, even though Bednar *et al*. have reported no differences in aggregation responses to TRAP in washed platelets between 25°C and 37°C in LTA (9). Other groups have, however, found that temperature does affect the responses to some agonists (15).

### Absorbance readings

Absorbance plate readers are ubiquitous to most labs and therefore the cost of this assay is greatly reduced compared to *de novo* setting up of LTA since there is no need to purchase dedicated equipment.

To gain accurate aggregation readings, a large signal range between the PRP and PPP absorbance readings is required, which is influenced by the absorbance wavelength. All reports are agreed on reading between 575 and 650 nm for PRP (9–11,15–17,20,23) which minimized absorption of plasma, and 405 nm for washed platelets (9,21).

The speed of the reading head of the reader does not seem to generally affect results. The introduction of stray bubbles in the wells, however, does greatly increase absorbance readings and produce erroneous aggregation values, hence reverse pipetting is recommended.

### Use of 96-well plate aggregometry in research and patients

Though 96-well plate aggregometry has been described by many groups, its primary use to date has been limited to research. The majority of this work concerns the characterization of platelet reactivity in the presence of antiplatelet therapies such as GPIIb/IIIa inhibitors (16,23) or dual anti-platelet therapy with ADP P2Y_12_ antagonists plus aspirin (10–13,17,21,22,25,26). Using these techniques, it is possible to produce concentration-response curves which can be used to evaluate the pharmacological action of these therapies. In addition to the *in vitro* exploration of these anti-platelet drugs, 96-well plate aggregometry has been used to describe *ex vivo* reactivity of platelets from patients with various diseases. Of note, enhanced platelet reactivity, particularly in response to ADP, has been described in patients with active inflammatory arthritis (27), HIV (28) and metastatic cancer (29).

Coupled with the study of platelet aggregation, other measures of platelet activation can also readily be assessed. For example, ATP release from dense granules after agonist stimulation can be detected using CHRONO-LUME® (Chronolog, Labmedics, UK) reagent. The firefly luciferase enzyme found in this reagent converts ATP to light which can be readily measured with standard laboratory equipment (30). In simple terms, the reagents can be added to a white-walled 96-well plate before mixing for a shorter period of time (2–3 minutes) and placing into a luminescent plate reader. This assay allows both laboratory-based studies into the mechanisms of granule release and the effects of drug therapies, as well as the clinical characterization of platelets from patients with suspected abnormal dense granule release.

## Lyophilized agonist platelet aggregation

Since 96-well plate aggregometry was first described, there have been few advances in the standardization of the method. To address this issue, we have developed a method to standardize the agonists and concentrations tested by producing standard plates at a centralized facility through the freeze-drying of agonists onto the plates. The lyophilization of the plates and subsequent vacuum packing produces plates that may be stored at room temperature for up to 12 weeks before use. We have also removed the variation in shaking type, speed and time by recommending a standard plate shaker as well as reducing the volume of PRP needed for each test to 40 μl (18,31).

Briefly, platelet agonists are lyophilized onto a standard half-area 96-well microtiter plate. The resulting ‘Optimul’ plate contains seven concentrations each of arachidonic acid (AA), ADP, collagen, epinephrine, ristocetin, TRAP-6 amide and U46619, but can be readily modified to include other reagents of interest (Figure 1C).

To perform the test, blood is taken into anti-coagulant and PRP and PPP isolated as per ISTH guidelines (6). About 40 μl of PRP or PPP is added to the appropriate wells of the Optimul plate and the plate is placed onto a BioShake IQ (QInstruments, Jena, Germany) thermomixer with 1200 rpm shaking at 37°C. The BioShake IQ has a planar orbital motion with a 2.0 mm diameter that generates a vortex within each well, imitating the dynamics of the LTA stir bar. After 5 minutes of shaking, the absorbance is read at 595 nm in any standard plate reader and the data are analysed through a fixed statistical and graphing protocol. From the generation of PRP to analysis, the assay takes less than 10 minutes.

A total of 2.5 ml PRP is needed to compare all agonist concentrations on the plate, which is the equivalent of approximately 6 ml blood compared to the approximate 50 ml that would be required to produce the same analysis through LTA (32). The low blood volume required makes this method of particular benefit in cases where blood sample is limited, such as in patients with advanced disease and in neonates.

### Use in research

The major disadvantage of this new technology is that there is a lack of widespread use and clinical data. We and others, however, have demonstrated that the effect of anti-platelet drugs can be elucidated using this method (18,31,32).

As with 96-well platelet aggregometry, release from platelet granules may also be studied in lyophilized agonist plates. Incubation of PRP with lyophilized agonists generates a concentration-dependent increase in ATP release as described earlier (18,30). Indeed, the supernatants from these tests may be used to detect levels of secondary mediators, such as TxA_2_, by ELISA. As the Optimul assay is conducted in standard 96-well plates, transferring samples to other assays is readily conducted using the very wide range of laboratory handling systems available for 96-well plates.

In addition to human platelet testing, a variation of the assay has also been used to phenotype platelet reactivity in whole blood of mice. The ultra-low volumes required make this method extremely suitable to phenotype platelet reactivity in whole blood of mice, particularly since a terminal blood collection from an individual mouse yields less than 1 ml of blood. Using a combination of Optimul to activate platelets in whole blood and flow cytometry to assess platelet populations, we have characterized platelet reactivity in genetically modified mouse strains and identified Intercellular Adhesion Molecule-1 (ICAM-1) as a novel mediator of platelet–monocyte interaction (33).

### Use in patients

An area for the potential application of the Optimul method is the diagnosis of bleeding disorders. Currently, particularly outside of dedicated centres, bleeding disorders attributed to platelet dysfunction are often poorly diagnosed because of the lack of specialized equipment for testing and operator inexperience. Because the Optimul method requires only standard equipment and the ability to prepare and pipette PRP, it may be easily run by routinely trained staff. In addition, a wider range of agonists and concentrations can be studied which provides a ‘global portrait’ of platelet responsiveness that may prove beneficial in diagnosing the 1% of the population with a mild bleeding disorder (32). Indeed, in collaboration we have compared LTA and Optimul aggregometry in samples from participants with suspected bleeding disorders within the Genotyping and Phenotyping of Platelets study and demonstrated that LTA was able to detect platelet defects in 54% of these people while the Optimul method was able to detect 66% (32).

Similarly, in two siblings with a 4-base-pair deletion in the *PLA2G4A* gene, resulting in the loss of group IV A phospholipase, Optimul was used to demonstrate a response to collagen-induced platelet aggregation similar to aspirin-treated healthy individuals but a preserved response to AA. These findings were confirmed by LTA (34).

### Population studies

Of particular interest is its use in population-based studies. To date, platelet reactivity has been rarely tested in large population studies due to the limitations and issues identified above, i.e. it is very difficult to maintain high throughput and low assay variation using LTA. Optimul aggregometry provides a solution in that a standardized test can be applied rapidly and consistently. Through collaborations, we are currently engaged in studying platelet reactivity through use of the Optimul method in the large cohorts of Bruneck and Framingham (300+ and 3000+ participants, respectively) to provide the first population-based studies of platelet reactivity.

## Conclusions

In conclusion, the Optimul method, with its ease of use, lack of need for specialized training, reduction in time and minimization of blood volume, opens the door to many possibilities for platelet testing in research and in the clinic. A major benefit of such a simple assay is its potential to study platelet reactivity in large populations who would otherwise not have routine platelet reactivity testing performed.
